# A novel variant of torque teno virus 7 identified in patients with Kawasaki disease

**DOI:** 10.1371/journal.pone.0209683

**Published:** 2018-12-28

**Authors:** James B. Thissen, Mariko Isshiki, Crystal Jaing, Yoshiro Nagao, Dayanara Lebron Aldea, Jonathan E. Allen, Masafumi Izui, Thomas R. Slezak, Takafumi Ishida, Tetsuya Sano

**Affiliations:** 1 Lawrence Livermore National Laboratory, Livermore, California, United States of America; 2 Department of Biological Sciences, Graduate School of Science, University of Tokyo, Tokyo, Japan; 3 Department of Pediatrics, Japan Community Health Care Organization Osaka Hospital, Osaka, Japan; University of Arizona, UNITED STATES

## Abstract

Kawasaki disease (KD), first identified in 1967, is a pediatric vasculitis of unknown etiology that has an increasing incidence in Japan and many other countries. KD can cause coronary artery aneurysms. Its epidemiological characteristics, such as seasonality and clinical picture of acute systemic inflammation with prodromal intestinal/respiratory symptoms, suggest an infectious etiology for KD. Interestingly, multiple host genotypes have been identified as predisposing factors for KD. To explore experimental methodology for identifying etiological agent(s) for KD and to optimize epidemiological study design (particularly the sample size) for future studies, we conducted a pilot study. For a 1-year period, we prospectively enrolled 11 patients with KD. To each KD patient, we assigned two control individuals (one with diarrhea and the other with respiratory infections), matched for age, sex, and season of diagnosis. During the acute phase of disease, we collected peripheral blood, nasopharyngeal aspirate, and feces. We also determined genotypes, to identify those that confer susceptibility to KD. There was no statistically significant difference in the frequency of the risk genotypes between KD patients and control subjects. We also used unbiased metagenomic sequencing to analyze these samples. Metagenomic sequencing and PCR detected torque teno virus 7 (TTV7) in two patients with KD (18%), but not in control subjects (P = 0.111). Sanger sequencing revealed that the TTV7 found in the two KD patients contained almost identical variants in nucleotide and identical changes in resulting amino acid, relative to the reference sequence. Additionally, we estimated the sample size that would be required to demonstrate a statistical correlation between TTV7 and KD. Future larger scale studies with carefully optimized metagenomic sequencing experiments and adequate sample size are warranted to further examine the association between KD and potential pathogens, including TTV7.

## Introduction

In 1967, Kawasaki disease (KD) was first reported as a pediatric illness with fever and systemic mucocutaneous inflammation [1]. KD is a vasculitic disease that causes a high rate of dilatation of coronary arteries [2] and may cause subsequent chronic cardiac complications [3]. Although the incidence of KD has increased in Japan and many other countries, its etiology remains unknown.

The incidence of KD presents with seasonality [4, 5]. KD is also often accompanied by prodromal intestinal and/or respiratory symptoms [6], and is preceded by contact with sick children [7, 8]. These characteristics suggest that infections might cause KD. In particular, the refractory response of KD to antibiotic therapy supports a viral etiology model [9], while the involvement of bacterial infections in KD pathogenesis is also hypothesized [10]. Although a correlation between viral infections and KD was reported [11, 12], these correlations were not reproduced in other studies [13–15]. Therefore, it remains to be elucidated whether the KD-associated infections were causal or incidental.

Importantly, KD incidence is much higher in populations with East Asian ancestry, in particular Japanese, compared to other ancestries [16]. This observation suggests a genetic propensity for KD among East Asian populations. Genetic surveys have identified variations at multiple loci as predisposing factors, including the genes for inositol 1,4,5-trisphosphate 3-kinase C (ITPKC)[17] and B-lymphoid tyrosine kinase (BLK)[18, 19]. ITPKC and BLK are related to immune regulation, suggesting that the pathophysiology of KD is closely linked to immune dysregulation. However, the high-risk alleles of single nucleotide polymorphisms (SNPs) are not necessarily more frequent in East Asian populations than in Caucasian populations; thus, the etiology of KD is still disputed [20]. Overall, it is widely assumed that one or more infections trigger an immunological reaction that leads to KD in genetically susceptible individuals [21].

In the present study, we aimed to explore experimental and epidemiological methodologies to identify an infectious cause for KD. In this report, we focused upon viral infections, whereas our analysis of bacterial infections in our samples will be reported elsewhere. Here, we propose a detailed protocol for metagenomic sequencing that would efficiently compare the microbial profiles between patients with KD and control individuals. We also demonstrate a possibility to study how interaction between the viral infection profile (i.e., the virome) and host genetic predisposition (particularly, ITPKC and BLK) might be involved in the development of KD. Our explorations resulted the identification of a new variant of torque teno virus 7 (TTV7) that was present only in two patients with KD.

In addition to effective experimental procedures, an epidemiological study design is essential for future large-scale studies to explore potential causative agent(s) of KD. The sample size is especially critical—by adopting an excessively large sample size, an investigation might detect a microbe of even negligible clinical importance as a potential culprit, whereas an inadequately small sample size might fail to detect a microbe that has a meaningful association with KD. Therefore, we estimated the sample size required to effectively demonstrate a statistically significant association between viral infections and KD.

## Results

### Participants

For a one-year study period, we enrolled 11 patients with KD and 22 control subjects (11 with diarrhea and 11 with respiratory infections) who were matched for sex, age and season of diagnosis (Table 1). As a result, we defined three groups of patients: 1) KD patients, 2) diarrhea controls, and 3) respiratory infection controls. The latter two groups constituted the control individuals. In terms of the number of KD-susceptible alleles of ITPKC or BLK per person, there were no statistically significant differences between the KD patients and the two control groups (Table 2).

**Table 1:** Profile of the patients with Kawasaki disease (KD) and the control subjects matched for sex, age, and season of diagnosis.

		KD		Diarrhea		Respiratory infections	
Seq.	Sex	Age (years)	Date	Age (years)	Date	Age (years)	Date
1	M	1.14	Sep. '14	1.07	Oct. '14	1.48	Sep. '14
2	F	3.33	Dec. '14	3.25	Jan. '15	3.68	Feb. '15
3	F	7.95	Jan. '15	4.70	Mar.' 15	5.29	Mar. '15
4	M	2.75	Jan. '15	2.98	Mar. '15	1.97	Feb. '15
5	F	2.28	Jan. '15	1.92	Mar. '15	2.59	Jan. '15
6	F	0.83	Jan. '15	1.34	Jan. '15	0.96	Feb. '15
7	M	4.19	Feb. '15	3.81	Jan. '15	3.69	Feb. '15
8	M	0.27	Feb. '15	0.87	Dec. '14	0.88	Feb. '15
9	M	0.75	May '15	1.03	Mar. '15	0.92	Apr. '15
10	M	3.47	Jun. '15	2.70	Jun. '15	3.05	Jun. '15
11	F	0.45	Jul. '15	1.08	Jul. '15	0.85	Jul. '15
Mean		2.49		2.25 (P^†^ = 0.859)		2.31 (P^†^ = 0.790)	

^†^P values, which correspond to the difference between KD patients and controls with diarrhea, or that between KD patients and controls with respiratory infections, were estimated by Wilcoxon's test.

**Table 2:** Genotypes of the SNPs of ITPKC (inositol 1,4,5-trisphosphate 3-kinase C) and BLK (B-lymphoid tyrosine kinase), which confer susceptibility to Kawasaki disease (KD).

	ITPKC rs28493229 (risk: C)			BLK rs2254546 (risk: G)			BLK rs2736340 (risk: T)		
Seq^††^	KD	Diarrhea	Respiratory infections	KD	Diarrhea	Respiratory infections	KD	Diarrhea	Respiratory infections
1	CC	GG	GG	GG	GG	AA	TT	TT	CC
2	GG	GG	GG	AG	GG	GG	CT	TT	CT
3	GG	GG	GG	GG	AA	GG	TT	CC	TT
4	GC	GG	GG	AA	AG	GG	CC	CT	TT
5	GG	GG	GC	GG	AG	GG	TT	CT	TT
6	GG	GG	GG	GG	AG	GG	TT	CT	TT
7	GG	GG	GG	GG	AG	AG	TT	CT	CT
8	GG	GG	GG	AG	GG	AG	CT	TT	CT
9	CC	GG	GC	AG	GG	GG	CT	TT	TT
10	GG	GC	GC	AG	AG	GG	CT	CT	TT
11	GG	GG	GG	AG	AG	GG	CT	CT	TT
Mean number of risk alleles	0.45	0.091 (P^†^ = 0.272)	0.27 (P^†^ = 0.587)	1.4	1.3 (P^†^ = 0.853)	1.6 (P^†^ = 0.330)	1.4	1.3 (P^†^ = 0.853)	1.5 (P^†^ = 0.477)

^†^P values were estimated by Wilcoxon's test.

^††^Seq corresponds to the sequence number in Table 1.

### Metagenomic sequencing of pooled samples

From each participant, we prepared several sample types, including serum, whole blood(WB) DNA, WB cDNA, nasopharyngeal aspirates (NPA), and feces. Half of each serum and NPA sample were treated with nuclease, to minimize any interference by the host DNA [22]. The samples were pooled according to sample type, within each of the three groups (i.e. KD, diarrhea, and respiratory infections). Pooled samples were used in metagenomic sequencing. After quality trimming, we obtained a total of 1,073,981,148 paired-end reads (accession number: DRA007000). We analyzed the dataset using the Livermore Metagenomic Analysis Toolkit (LMAT) [23–25]. After computational subtraction of human genomic sequences, we mapped 6,270,435 reads to microbial sequences. Specifically, we mapped 5,301,257 reads (85%) to bacteria (154 families, 460 genera, 1,644 species); 594,900 reads (9.5%) to viruses (16 families, 31 genera, 69 species); and 374,278 reads (6.0%) to eukaryotes.

### Virome

**Fig 1:**
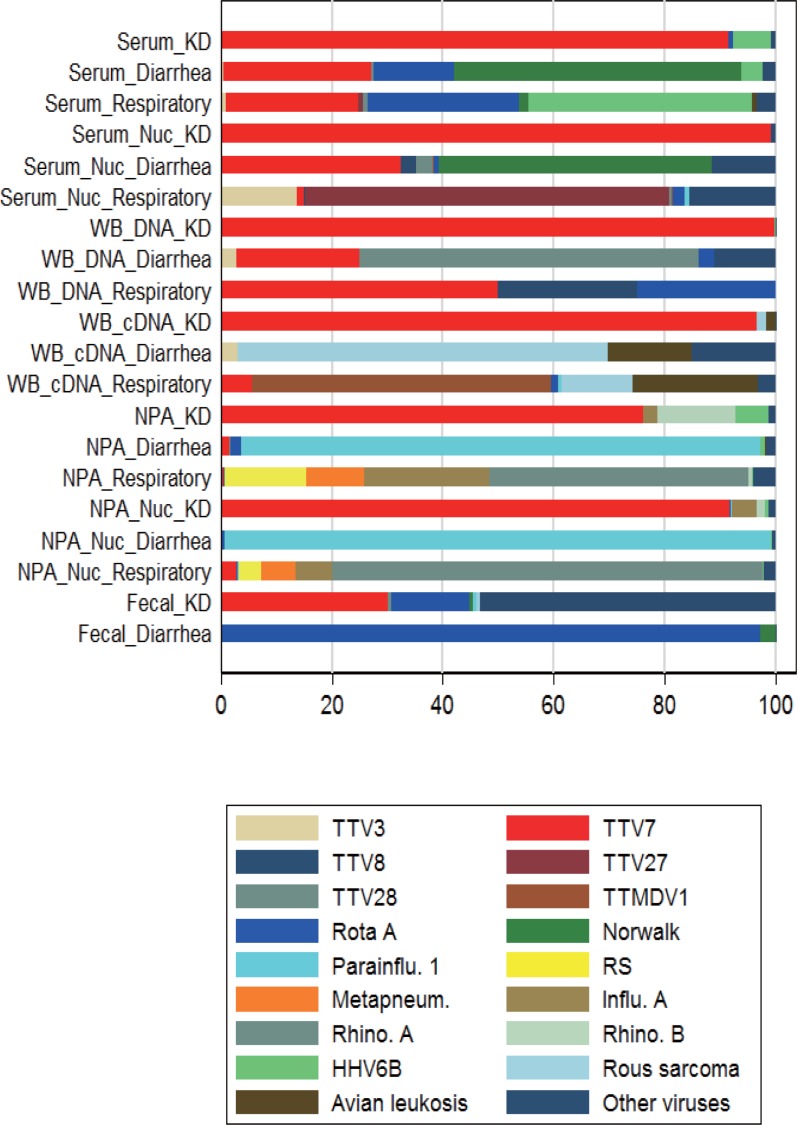
Composition of reads mapped to viral species (virome) in each pooled sample. The three most abundant species in each pooled sample type are included. Numerical data are available in S1 Table. Kawasaki disease, KD; whole blood, WB; nasopharyngeal aspirate, NPA; nuclease treatment, Nuc; torque teno virus, TTV; torque teno midi virus, TTMDV; rotavirus A, Rota A; human parainfluenza virus 1, Parainflu 1; respiratory syncytial virus, RS; influenza virus A, Influ. A; human metapneumovirus, Metapneum; rhinovirus, Rhino; human herpesvirus 6B, HHV6B; rous sarcomavirus, Rous sarcoma; avian leukosisvirus, Avian leucosis.

Table 3 shows the number of reads, classified as either viruses or bacteria, in each of the pooled samples. Nuclease treatment resulted in a much greater number of reads. Viral reads constituted the majority of the microbial reads in serum with and without nuclease processing, WB DNA, and WB cDNA, pooled from the KD patients. Fig 1 shows the composition of reads mapped to viral species (i.e. virome) in each sample (S1 Table). Among these viral species, torque teno virus 7 (TTV7) constituted the vast majority of reads in any of the sample types pooled from KD patients, but not in samples pooled from the control subjects.

**Table 3:** Number of reads mapped to viral or bacterial species in the pooled samples.

Sample type	Viral reads	Bacterial reads	% Viral reads in all microbial reads
Serum_KD	131	85	61
Serum_Diarrhea	181	506	26
Serum_Respiratory	117	1,103	9.6
Serum_Nuc_KD	119,526	13,900	90
Serum_Nuc_Diarrhea	7,143	223,129	3.1
Serum_Nuc_Respiratory	834	38,753	2.1
WB_DNA_KD	37,084	757	98
WB_DNA_Diarrhea	36	119	23
WB_DNA_Respiratory	4	460	0.86
WB_cDNA_KD	100,447	15,405	87
WB_cDNA_Diarrhea	33	24,843	0.13
WB_cDNA_Respiratory	163	21,860	0.74
NPA_KD	248	82,574	0.30
NPA_Diarrhoea	740	13,891	5.1
NPA_Respiratory	474	23,300	2.0
NPA_Nuc_KD	2,937	403,688	0.72
NPA_Nuc_Diarrhea	31,379	67,336	32
NPA_Nuc_Respiratory	1,837	40,422	4.4
Fecal_KD	143	2,273,391	0.01
Fecal_Diarrhea	291,443	2,055,735	12

The abbreviations in the sample types are: Kawasaki disease, KD; Nuc, treatment with nuclease; nasopharyngeal aspirate, NPA; whole blood, WB.

### Variants of TTV7

**Fig 2:**
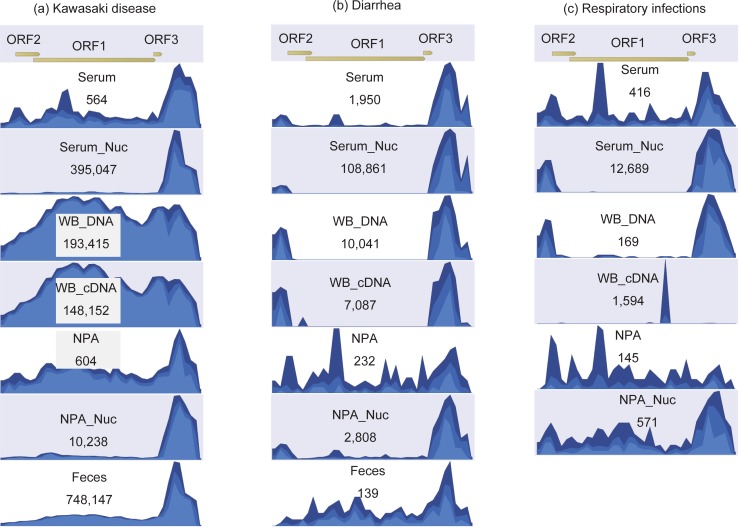
Depth of reads mapped to the reference genome of torque teno virus 7 (TTV7) in the pooled samples. Reads pooled from Kawasaki disease (a), controls with diarrhea (b), and controls with respiratory infection, were mapped to the TTV7 reference sequence (AF261761). Reads were extracted from diverse sample types, including serum, serum with nuclease treatment (Serum_nuc), DNA from whole blood (WB_DNA), complementary DNA converted from WB_DNA (WB_cDNA), nasopharyngeal aspirates (NPA), NPA with nuclease treatment (NPA_Nuc), and feces.

The reads from metagenomic sequencing were mapped to the reference sequence of TTV7 (Fig 2). For all sample types, the reads obtained from KD patients were mapped to both the coding region [i.e. open reading frame (ORF)] and the non-coding region. However, in the control subjects, the reads were mapped only to the non-coding regions in most of the sample types. This finding suggests that TTV(s) other than TTV7 exist in the control subjects, because non-coding regions are shared by multiple TTV strains, whereas only the ORFs are specific to individual TTV strains (S1 Fig). To identify anelloviruses other than TTV7, we mapped the reads to all human anelloviruses: TTV1 to 29, torque teno mini virus (TTMV) 1 to 9, and torque teno midi virus (TTMDV) 1 and 2. We found that the WB DNA of all three groups (i.e. KD, diarrhea, and respiratory infection) contained multiple TTVs (S2 Fig).

### PCR and Sanger sequencing of TTV7

**Fig 3:**
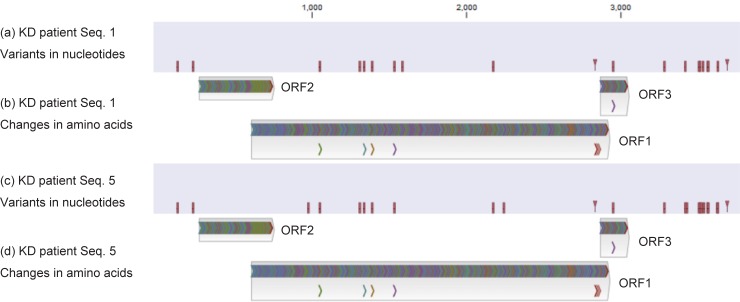
Variants in nucleotides and amino acids of the TTV7 determined by Sanger sequencing of individual Kawasaki disease (KD) patients. Sanger sequencing was applied to TTV7 in KD patient seq. 1 (Table 1) and revealed variants in nucleotides (a), which resulted in amino acid changes (b). Similarly, TTV7 in KD patient seq. 5 harbored variants in nucleotides (c), which changed amino acids (d). The amino acid changes are identical between the two TTV7s. Spread sheet data is available in S2 Table. Splicing is not considered in the analysis of amino acid changes.

Using PCR, we detected TTV7 sequence in WB DNA, NPA, and feces from two KD patients (seq. 1 and seq. 5 in Table 1), but not in samples from the control individuals (Table 4). We used Sanger method to sequence the TTV7 genome from the two KD patients. While the reference sequence was 3,736 base pairs in length, we sequenced 3,699 base pairs, excluding the GC-rich region. As a result, we found that these two DNA sequences were very similar (Fig 3A and 3C, S2 Table). The sequences of these TTV7s are available at DNA Data Bank of Japan (accession numbers: LC385662, LC385663). The translated amino acid sequences resulting from these two TTV7 sequences were identical (Fig 3B and 3D, and S2 Table). In addition, it is possible that the changes in the nucleotide sequence affected the ORFs in these TTV7s (S3 Fig). Considering the complex processes of replication in TTVs (especially, alternative splicing) [26], the impact of these mutations may not be limited to these amino acid changes.

**Table 4:** Presence of TTV7 revealed by metagenomic sequencing and polymerase chain reaction (PCR) of the individual samples.

	Kawasaki disease				Diarrhea				Respiratory infections		
Seq.	Metagenomic sequencing	PCR			Metagenomic sequencing	PCR			Metagenomic sequencing	PCR	
	WB	NPA	WB	Feces	WB	NPA	WB	Feces	WB	NPA	WB
1	+	+	+	+	–	–	–	–	–	–	–
2	–	–	–	–	–	–	–	–	–	–	–
3	–	–	–	NA	–	–	–	NA	–	–	–
4	–	–	–	NA	–	–	–	NA	–	–	–
5	+	+	+	NA	–	–	–	NA	–	–	–
6	–	–	–	–	–	–	–	–	–	–	–
7	–	–	–	NA	–	–	–	NA	–	–	–
8	–	–	–	–	–	–	–	–	–	–	–
9	–	–	–	–	–	–	–	–	–	–	–
10	–	–	–	NA	–	–	–	NA	–	–	–
11	–	–	–	–	–	–	–	–	–	–	–

Fecal samples were not available for subjects with respiratory infections. NA, not available. Seq corresponds to the sequence number in Table 1.

### Metagenomic sequencing of individual samples

Because the reads mapped to TTV7 from WB DNA samples were the most homogeneous and the greatest in number (Fig 2), we employed WB DNA for the metagenomic sequencing of individual subjects (n = 33). Each individual sample yielded 13,387,302 reads on average (range: 11,593,598–14,805,260 reads), of which 93–94% were human sequences. The LMAT analysis on these datasets reported more than 10 viral reads for two viruses, which were found in only three individuals: TTV7 in two KD patients (seq. 1 and 5 in Table 1) and Human Herpes Virus 6B (HHV6B) in one KD patient (seq. 8).

**Fig 4:**
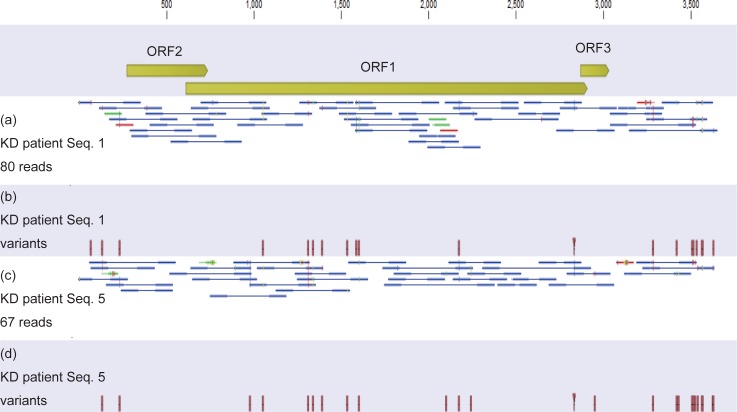
TTV7 in individual whole blood (WB) DNA samples, identified by metagenomic sequencing. Metagenomic sequencing was applied to the WB DNA samples from the 33 individuals. Reads from Kawasaki disease (KD) patient seq. 1 were mapped to the TTV7 reference genome (a) and showed variants (b). Similarly, the reads from KD patient seq. 5 were mapped to TTV7 (c) and generated variants (d). Although the depth was shallow, the reads covered the entire genome seamlessly (a, c). The variants (b, d) resembled the variants determined by Sanger sequencing (Fig 3), with limited accuracy due to the shallow depth.

We mapped the metagenomic sequencing reads to these viruses and to all human anelloviruses. As shown in Fig 4, we detected TTV7 in the two KD patients (seq. 1 and 5), but not in the control subjects (Table 4). The depth of reads mapped to human genome was 0.42 per base in average, while the depth of reads mapped to TTV7 genome in the two KD patients was 3.0, demonstrating the abundance of TTV7. On the other hand, the depth of reads mapped to HHV6B genome (>160,000 bases) in the KD patient seq. 8 was only 0.078, indicating that HHV6B may not be present in this sample. Therefore, TTV7 was the only virus that was unambiguously present. This decreased sensitivity, when compared to the metagenomic sequencing of the pooled samples, was possibly due to the whole transcriptome amplification that we used only for the pooled samples but not for the individual samples (please see the Methods section). In summary, this result demonstrates that TTV7 had a much higher viral load in the peripheral blood than other viruses.

### Re-evaluation of pooled samples

**Fig 5:**
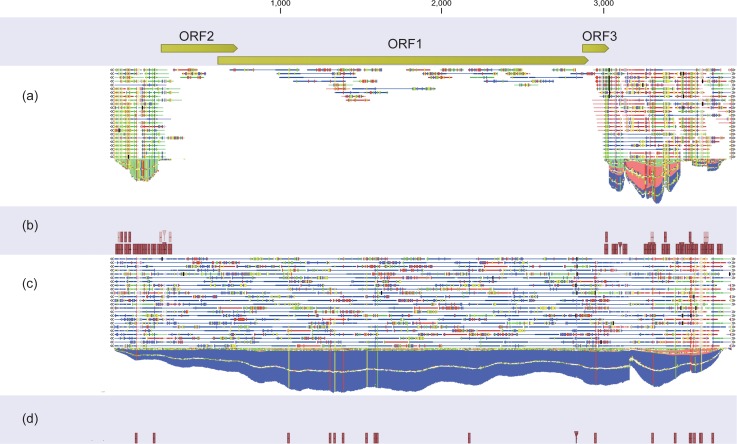
Whole Blood (WB) DNA pooled from diarrheal control individuals and that from patients with KD were mapped to the reference sequence of TTV7. In the diarrheal control individuals, 10,041 reads mapped to TTV7, of which only 40 reads (0.40%) mapped to the ORFs of TTV7 (a). The reads from the diarrheal control individuals did not contain the variant nucleotides identified in the present report (b). However, in the patients with KD, 193,415 reads mapped to TTV7, of which 156,566 reads (81%) mapped to the ORFs of TTV7 (c). The reads pooled from the patients with KD contained the variant nucleotides (d).

In metagenomic sequencing of the pooled samples, some reads from the control individuals apparently mapped to TTV7. For example, a considerable number of reads from the pooled WB DNA of the control diarrheal patients appeared to map to TTV7 (Fig 1). To re-evaluate whether TTV7 existed in the control diarrheal patients, we mapped the reads in the pooled WB DNA of the control diarrheal patients to the TTV7 reference genome. Of the 10,041 reads that mapped to TTV7, only 40 reads (0.4%) were mapped to the ORFs of TTV7 (Fig 5A). Because only the ORFs of TTV7 are strain specific (S1 Fig), this finding implied that almost all of the reads that appeared to map to TTV7 in fact originated from TTVs other than TTV7. In addition, the 40 reads that mapped to the ORFs of TTV7 did not contain any of the variant nucleotides that we identified in this report (Fig 5B). In contrast, among the 193,415 reads from the pooled WB DNA of the patients with KD that mapped to TTV7, 156,566 reads (81%) mapped to the ORFs of TTV7 (Fig 5C) and contained the variant nucleotides (Fig 5D). We repeated this bioinformatic analysis for other types of samples and confirmed that the variant TTV7 was not present in the control individuals. Therefore, the apparent identification of TTV7 in the control individuals was most likely erroneous because closely related strains of TTVs were simultaneously present in those individuals [27].

### Statistical analysis

Two of 11 KD patients were positive for TTV7 sequence, whereas all 22 controls were negative (P = 0.104, by Fisher's exact test). Because the samples were matched, we employed exact logistic regression analysis to estimate the dependence of KD on the presence of TTV7 and the number of risk alleles of ITPKC and BLK in each individual. Due to the small sample size, we did not conduct a multivariate analysis. As shown in Table 5, we found that dependence of KD on the presence of TTV7 was not significant, with an odds ratio (OR) of 4.8 (P = 0.111). The dependences of KD on ITPKC and BLK were also not significant, with weaker ORs.

**Table 5:** Odds ratios which represent correlation between Kawasaki disease and TTV7 or host genotype, estimated by exact logistic regression.

	Odds Ratio (OR)	95% confidence interval of OR	P value
Presence of TTV 7	4.83	0.376 –Positive Infinity	0.111
Number of risk alleles of ITPKC rs28493229	3.82	0.607–138	0.161
Number of risk alleles of BLK rs2254546	0.856	0.294–2.57	0.812
Number of risk alleles of BLK rs2736340	0.925	0.317–2.81	1.00

### Estimation of sample size

Table 6 shows the sample size that would be necessary to detect a statistically significant association between TTV7 and KD. We also conducted a sensitivity analysis based upon multiple assumptions regarding the prevalence of TTV7 in the control population.

**Table 6:** Estimated number of cases of Kawasaki disease that would be necessary to demonstrate statistically significant correlation between KD and TTV7.

Number of matched controls per case of KD	1	2	3	4
Prevalence of TTV7 in control subjects.				
5%	102	75	66	62
2%	223	164	145	135
1%	426	313	276	258

We adopted standard statistical assumptions for an epidemiological study (i.e., α level of 5% and β level of 90%).

## Discussion

We applied metagenomic sequencing to samples pooled from patients with KD, as well as from control subjects. We found that most of the sequencing reads in blood samples pooled from patients with KD were of viral origin, and the vast majority of these viral reads were TTV7. We also detected a variant of TTV7 in two patients with KD, whereas all of the matched control subjects were negative for this variant TTV7. Notably, the nucleotide sequences of TTV7 in these two KD patients were almost identical. Our small pilot study found only a marginal, non-significant correlation between KD and TTV7, which was comparable to the correlations between the host genotypes and KD.

TTV was first identified by Nishizawa et al. in 1997 [28]. Subsequently, TTV and newly identified TTV-related viruses formed a novel viral family, *Anelloviridae* [29]. Currently, the *Anelloviridae* family contains three human viral genera (TTV, TTMV, and TTMDV) and an expanding list of non-human genera. The pathogenicity of anelloviruses remains unknown, except for minor respiratory symptoms and fever in children [30, 31]. Among the TTVs, TTV7 was reported in 2000 [32].

In our study, the anellovirus TTV7 was the most abundant virus that was present in feces, upper airway, and blood of two KD patients. This pantrophic characteristic of TTV7 is consistent with the intestinal and respiratory symptoms that are prodromal to KD. Previous epidemiological studies predicted that at least one of the etiological agent(s) for KD would be capable of persistent infection [33, 34]. Notably, persistent infection is a characteristic reported to be shared by the anelloviruses [35].

Furthermore, it was mathematically predicted that the infectious etiological agent of KD would be relatively common [34], based upon the young mean age of KD patients in Japan [36]. Consistent with this line of thought, TTVs are highly prevalent among healthy adult individuals [37]. However, TTV7 was reported to be rare [38]. In our study, TTV7 was detected in only two individuals among the 33 participants. The relative rarity of TTV7 is not consistent with epidemiological predictions for a common etiological agent of KD. Therefore, we must be cautious before hypothesizing that TTV7 is the sole etiological agent of KD. Instead, we hypothesize that TTV7 may be one of the multiple agents contributing to KD etiology.

TTVs have often appeared in the etiological studies of KD. For example, TTV was the only virus detected by PCR in a lymph node biopsy from a KD patient [39]. In contrast, an early anellovirus research study did not find a statistical correlation between TTV and KD [14]. However, we found that the PCR primer sequences used in this early study [14] did not capture many strains of TTV, including TTV7. This lack of sensitivity was because these early PCR primers did not account for the diverse variation in TTVs. Although recent degenerative PCR primers are more inclusive of this diversity [37], the differentiation between the TTV genotypes as well as the quantification of their viral loads remain a challenge for the field. In contrast, the metagenomic sequencing methodology, which was used for the pooled samples in this study, provides a relative quantification of viral loads in the form of number of reads and allows for differentiation between strains of TTV. However, the subsequent metagenomic sequencing method that we employed for the individual samples was not very sensitive. This limitation highlights the importance of careful optimization of sequencing methods that is specific for the sample types and the targeted viruses. We also estimate that a larger sample size will be necessary to yield a statistically significant result in a future etiological study of KD. Even though our sample size of KD patients was small, we could enroll the matched control subjects only with the assistance of an in-house software. This predicts the challenge in enrolling matched control subjects in a future study.

Hypothetically, the discovery of infectious etiological agent(s) should be confirmed by a temporal association between the infection and the disease (i.e., an infection with the etiological agent should precede development of the disease). That criterion was not met, however, in almost all previous etiological discoveries. Rather, statistical correlation between the presence of an infectious agent and the development of disease was employed to suggest etiology, at least in the early stage [40]. To prove a temporal association between a viral agent and KD, it would be necessary to collect samples sequentially from healthy infants until either they develop KD or they grow too old to develop KD (e.g., at 20 years of age). Considering the probability that a Japanese child will experience KD at some time in his or her life (1% of all children), such a study design would require a huge number of participants (e.g., 10,000 healthy infants phlebotomized regularly for 20 years to identify 100 patients with KD). The metagenomic sequencing of such a large number of samples would be cost-prohibitive. An alternative approach is an animal model. It might be possible to study the etiology of KD by administering a candidate microbe (e.g., TTV7) into mice and observing the resulting pathology in the coronary arteries [41–43]. Most microbes, including TTVs, are adapted to their natural host species, hence often induce exaggerated immunological responses in species other than their natural host. Therefore, studies using animal models may have limitations.

Our pilot study provides insights to inform future investigations of KD etiology. The sample size predicted from our study, the experimental procedures and data analysis methods will benefit future large-scale epidemiological studies. These larger scale studies may confirm TTV7 and discover additional microbial agents that could contribute to the etiology of KD. Studies with animal models might also contribute to the elucidation of the pathological process caused by these candidate microbes.

## Materials and methods

### Ethical statement

This study was approved by the ethics committee of Japan Community Health Care Organization (JCHO) Osaka Hospital. Written informed consent was obtained from the guardians of the participants.

### Participants

Between October 2015 and September 2016, we enrolled 11 patients with KD. The diagnosis was based upon the criteria of Japanese Circulation Society [44]. We found that it was impractical to use completely healthy individuals as controls because healthy children rarely visit a hospital. Therefore, we enrolled febrile individuals as controls. In enrolling matched control individuals, we adopted a 1:n (n>1) design, because that approach increases the statistical power as compared with a 1:1 design. However, the controls assigned to each patient with KD should be homogeneous across all the patients with KD: otherwise, nonhomogeneous controls might induce biases and thus generate unpredictable results. To achieve homogeneity among the control individuals, for each patient with KD, we enrolled two febrile control subjects: one with diarrhea and the other with respiratory infection. The control subjects were matched to a patient with KD by gender, age, and season of diagnosis. The difference in the age between a KD patient and the matched control subject was maximally 0.8 year, if the KD patient was younger than 4.5 years. For older patients with KD, the control subjects were also selected from children older than 4.5 years. The difference in the time-point at presentation between a KD patient and the matched control subject was no greater than 2 months. We developed a software to assist in selecting the candidates of control subjects on the daily basis.

We defined a patient of respiratory infection as a presentation of sore throat, cough, rales, and/or wheezes. We excluded diarrhea of non-infectious origin (e.g. anaphylaxis, drug-induced) from the control subjects with diarrhea. All the control subjects were either febrile (>38.0°C) at presentation, or within 24 hours after defervescence. Subjects with conjunctivitis, rash, and/or lymph-node enlargement were excluded from the control group. We collected samples from KD patients before initiating treatment with intravenous immunoglobulin.

### Sample collection

Peripheral blood was phlebotomized in maximally sterile conditions and the serum was immediately aliquoted. One sterile swab collected nasal aspirate, while another swab collected pharyngeal aspirate. These two swabs were stored in a single Universal Transfer Medium tube (COPAN, Brescia, Italy) to produce NPA. Feces were also collected into another Universal Transfer Medium tube. We obtained feces from only two groups (six KD patients and six matched diarrheal patients). Aliquoted serum, blood clot which remained after separation of serum, and NPA were frozen within 1 hour after sample collection, whereas feces were frozen within 6 hours. The samples were preserved at –80°C until being used.

### Preparation of DNA and cDNA from WB

To identify the host genotype and to detect the microbial genomes in the peripheral blood cells, we extracted DNA and RNA from the blood clot. Subsequently, the RNA was converted to cDNA. We used a High Pure PCR Template Preparation Kit (Roche, Penzberg, Germany) for DNA extraction and a NucleoSpin RNA Blood kit (MACHEREY-NAGEL GmbH & Co. KG, Gueren, Germany) for RNA extraction. We immediately converted the extracted RNAs to cDNA with a Transcriptor First Strand cDNA Synthesis Kit (Roche, Penzberg, Germany). To synthesize cDNA, the RNA was denatured in a 13 μl cocktail containing 10 μl total RNA, 2 μl Random hexamer primers, and 1μl Anchored oligo (dT)18 primer at 65ºC for 10 minutes. Then, we added 0.5 μl Protector RNase Inhibitor, 0.5 μl Transcriptor Reverse Transcriptase, 2 μl dNTP Mix and 4 μl Transcriptor RT Reaction Buffer to the reaction mix. Finally, the reverse transcription reaction was performed in this 20 μl cocktail using the following conditions: pre-incubation at 25°C for 10 minutes, incubation at 50°C for 60 minutes, inactivation of Transcriptor Reverse Transcriptase at 85°C for 5 minutes, and storage at 4°C.

### Host genotyping

To genotype rs2254546 and rs2736340 in the BLK [18, 19], as well as rs28493229 in the ITPKC [17], we obtained a DNA fragment of 480 base pairs containing rs2254546 and rs2736340 and another fragment of 202 base pairs containing rs28493229 by PCR using the following two primer sets, respectively: 5’-CCACGGAGAAAACTGCTGGA-3’ (forward primer) and 5’-AGAGGTGCCATTTCTGGGTG-3’ (reverse primer); 5’-GAGTCTGAGGATGACGTGGTG-3’ (forward primer) and 5’-CAGTGGATGGAAGAGGTTCCC-3’ (reverse primer). All PCR amplifications were performed in a 12.5 μl cocktail containing 1–2 μl genomic DNA, 0.25 μl of each primer (10 μM), 1.25 μl of 10× PCR Buffer containing 15 mM MgCl_2_, 1.25 μl of 2 mM dNTP mixture, and 0.125 μl AmpliTaq Gold DNA Polymerase (Thermo Fisher Scientific, Waltham, MA). The following cycling conditions were used: pre-incubation at 95°C for 10 minutes; 35 cycles of 30 seconds at 95°C, 30 seconds at 58°C, and 30 seconds at 72°C; and final extension for 7 minutes at 72°C. The PCR products were purified with a Favor Prep PCR Clean-Up Mini Kit (Favorgen, Ping-Tung, Taiwan). Purified PCR products were sequenced bi-directionally by Eurofins Genomics (Tokyo, Japan).

### Preparation of nucleic acids from serum, NPA, and feces

An equal amount (450 μl) of the individual serum and NPA samples were placed in a pre-wet 0.45 μm Spin-X Tube Filters (Corning, Corning, NY), and centrifuged for 1 minute at 15,000 × g. The flow-through was pooled in each of the three groups (KD, diarrheal control, and respiratory infection control). The supernatants were ultra-centrifuged at 4ºC for 2 hours at 25,000 × g to pellet the virus particles. We then removed the supernatant and resuspended the pellet in 400 μl of 1× AM2238 Turbo DNase buffer (Ambion, Foster city, CA). Previously, it was reported that nuclease is effective in eliminating host nucleotides [22]. Therefore, we processed half of the resuspended pellet without nuclease, whereas the other half was processed with nuclease. We added four nucleases to the sample: 1 μl Benzonase 70664–3 (EMD, Darmstadt, Germany), 1 μl Baseline Zero DB0711K (Epicentre, Madison, WI), 1 μl AM2238 Turbo DNase (Ambion), and 1μl A7973 RNase A (Promega, Madison, WI), then incubated at 37°C for 90 minutes. Nucleases were inactivated by addition of 2 μl of Baseline Zero DNase Stop Solution (Epicentre) and incubation at 65°C for 10 minutes.

We used the QIAamp cador Pathogen Kit (QIAGEN, Hilden, Germany) for each set of serum and NPA samples (with and without nuclease treatment) following the manufacturers’ protocol, to allow for maximal retrieval of viral DNA/RNA while minimizing the presence of bacterial/host DNA/RNA. The DNA/RNA was resuspended at the final step by adding 50 μl of Buffer AVE (QIAGEN,) to each QIAamp Mini column (QIAGEN). After centrifugation at full speed (20,000 × g), we collected the elute (50 μl).

The MoBio Powerviral Environmental RNA/DNA Kit (MoBio, Carlsbad, CA) was utilized for the individual fecal samples. Briefly, 0.25 grams of fecal material was added to bead lysis tubes and the nucleic acids were extracted following the manufacturers’ protocols. Purified nucleic acids were eluted in 50 μl of RNase-free water.

We performed the reverse transcription reaction with the following reagents: SuperScript III Reverse Transcriptase (Invitrogen, Carlsbad, CA), RNaseOUT Recombinant Ribonuclease Inhibitor (Thermo Fisher Scientific), 100 mM dNTP Set (Invitrogen), 5’-phosporylated random hexamer primer (Integrated DNA Technologies, San Jose, CA), Quantitect Whole Transcriptome Kit (QIAGEN), and QIAquick PCR Purification Kit (QIAGEN). The reaction consisted of: sample (10 μl), RT Primer (5’-phosphorylated random hexamers; 1 μl of 250 ng/μl), and dNTP mix (1 μl of 10 mM). We mixed these components and incubated the reaction at 85°C for 5 minutes, then cooled it on ice. Then, we added 5× Superscript III buffer (4 μl), Dithiothreitol (1 μl of 0.1M), RNaseOUT Recombinant Ribonuclease Inhibitor (Thermo Fisher Scientific) (1 μl of 40 U/μl), Superscript III RT (1 μl), and Diethyl pyrocarbonate water (1 μl) to the reaction and incubated at: 25°C for 10 minutes, 42°C for 60 minutes, 95°C for 5 minutes, then held on ice.

### Whole transcriptome amplification of nucleic acids pooled from serum, NPA, feces, WB-DNA, and WB-cDNA

The whole transcriptome amplification optimally amplifies viral nucleic acids [22]. Therefore, we applied the whole transcriptome amplification to the nucleic acids pooled from serum, NPA, feces, WB-DNA, and WB-cDNA. WTA ligation buffer (6 μl), WTA ligation reagent (2 μl), WTA ligation enzyme 1 (1 μl), and WTA ligation enzyme 2 (1 μl), which are all provided in the REPLI-g Cell WGA and WTA Kit (QIAGEN), and the pooled nucleic acids (10 μl) were mixed and incubated at 22°C for 2 hours. Then, REPLI-g Midi reaction buffer (29 μl) and RFPLI-g Midi DNA polymerase (1 μl), also provided in the above kit, were added and incubated at: 30°C for 8 hours, 95°C for 5 minutes, then held on ice. The PCR product was cleaned using the QIAquick PCR Clean-up kit (QIAGEN) following the standard manufacturer’s protocols.

### Metagenomic sequencing of the pooled samples

We prepared DNA libraries for sequencing using the TruSeq PCR-free DNA Library Preparation Kit (Illumina, San Diego, CA). Quality and fragment size were assessed on the Tapestation (Agilent, Santa Clara, CA). Libraries were normalized to 2 nM, pooled, denatured, and diluted to 1.8 pM, according to the manufacturer’s standard recommendations (Illumina). Sequencing was performed on the NextSeq 500 with the NextSeq Series High Output Kit version 2 (Illumina), using 150 base pair, paired-end reads.

### Metagenomic sequencing of the individual samples

The WB DNAs from the 33 individual participants, without the whole transcriptome amplification, were outsourced to Hokkaido System Corporation, where TruSeq Nano DNA Library Prep kit (Illumina) was used for the metagenomic sequencing. We omitted the whole transcriptome amplification because TruSeq Nano DNA Library Prep kit includes a PCR step, and therefore allows for a lower input concentration. Quality and fragment size were assessed on BioAnalyzer 2100 (Agilent), and the concentrations were determined by quantitative PCR. The libraries were normalized to 4nM, pooled, denatured, and diluted to 14 pM, following the manufacturer's recommendation (Illumina). Sequencing was performed on the HighSeq 2500 with HiSeq Rapid PE Cluster Kit v2 and HiSeq Rapid SBS Kit v2 (Illumina), using 100 base pair, paired-end reads.

### Bioinformatic analysis

The metagenomic sequencing data was analyzed using the LMAT [23–25]. LMAT is a metagenomic analysis pipeline to search for taxonomic identifiers associated with k-mers found in the reference genome database. The database contains 4,863 distinct bacterial species; 4,189 distinct viral species; 2,038 distinct eukaryotes; and 279 distinct archaea species. Each read was assigned a match score, which is the fraction of k-mers in the read that were matched to the corresponding reference genome divided by a randomly generated read with similar GC content and a comparable length. We discarded any read with a match score below the designated threshold that is expected by the null model. A match score threshold of 1 represents the percent identity match of double what would be observed by a random match, resulting in stringent species identification. A threshold of 0.5 is more permissive. Using 1 or 0.5 for the threshold did not qualitatively affect our results and conclusion, and we presented the results obtained by the threshold of 1.

CLC Genomics Workbench 10.1.1 (QIAGEN) was used to visualize the depth and extent of the reads mapped to the reference genome. This software was also used for variant analysis. We employed the accession numbers defined in ref. [29] as the reference sequences for TTVs.

### Statistical analysis

Stata SE 13.1 (StataCorp, College Station, TX) was used for statistical analyses. Conditional regression analysis could not be used to assess the effect size (i.e., odds ratio), because the point estimate of the odds ratio reached an infinite positive value. Instead, we used exact logistic regression analysis. In estimating sample size, we assumed a standard statistical requirement for an epidemiological study (i.e. α level of 5% and β level of 90%).

### PCR and Sanger’s sequencing of TTV7

We amplified the whole genome of TTV7 using two primer sets (S4 Fig): 5’-CCCCGGTATACAATGTCCCG-3’ as the forward primer and 5’-CTCCGTGGTATGCAAAGGGT-3’ as the reverse primer (primer set 1); 5’-AAAGCATCGTGAGCATGCAG-3’ as the forward primer and 5’-CAGACGGCAACGGGTACATA-3’ as the reverse primer (primer set 2). Each of these PCR amplifications were performed in a 5 μl reaction mixture, containing 1.0 μl genomic DNA, 2.5 μl 2× PCR buffer for KOD-FX (Toyobo, Osaka, Japan), 1.0 μl 2 mM dNTPs, 0.075 μl 20 mM forward and reverse primers, 0.25 μl DDW, 0.25 μl 2.0 μg/μl bovine serum albumin (BSA), and 0.1 μl KOD-FX. We used the following cycling conditions: pre-incubation at 94°C for 2 minutes 40 cycles of 10 seconds at 98°C, 2 minutes at 68°C; and a final extension at 68°C for 7 minutes. PCR amplification was examined by electrophoresis on 1% agarose gel. The PCR products were purified with FavorPrep PCR Clean-Up Mini Kit (Favorgen, Ping-Tung, Taiwan). Purified PCR products were directly sequenced using the above two primer sets in Eurofins Genomics (Tokyo, Japan). The PCR products amplified with the primer set 2 were also sequenced using newly designed two primers, 5’-GGGCAAGGCTCTTAGGGTTAT-3’ and 5’-TAAACAAGGCCGTGGGAGTT-3’. Sequencing was performed more than twice. We aligned the obtained sequences manually and by Clustal W [45], implemented in MEGA version 6 [46], using the published TTV7 sequences as references (GenBank accession number: AF261761).

## Supporting information

S1 Fig Torque teno viruses (TTVs) were fragmented and mapped to TTV7. The genomes of TTVs, other than TTV7, were fragmented into 80 bases and mapped to the genome of TTV7. These fragments were mapped only to non-coding regions but not to open reading frames (ORFs). This indicates that the ORFs are specific to the strain of TTVs, while the non-coding regions are shared by the strains.
(EPS)

S2 Fig Strains of TTVs in the pooled whole blood (WB) DNA samples identified by metagenomic sequencing. Reads from pooled WB DNA samples were mapped to anelloviruses. WB DNA pooled from Kawasaki disease (KD) patients was mapped to TTV5 (a) and TTV15 (b), that from diarrhea controls was mapped to TTV15 (c) and TTV29 (d), and that from respiratory infection controls was mapped to TTV5 (e) and TTV22 (f). The reads were mapped to each genome, indicating the presence of the virus.
(EPS)

S3 Fig Open Reading Frames (ORFs) predicted for TTV7s. ORFs were predicted in the reference sequence of TTV7 (a), and in the TTV7s identified in two KD patients (b, c). Minimal length of an ORF was assumed to be 50 bases. To date, experimental studies showed that three ORFs (ORF1, 2 and 3) were involved in the protein synthesis.
(EPS)

S4 Fig The design of primer sets which were used for PCR of TTV7. The primer set 1 was designed to span from the open reading frame (ORF) 2 to ORF1, while the primer set 2 covered the rest of the circular genome of TTV7.
(EPS)

S1 Table Abundance of reads mapped to viruses in the pooled samples (numerical data for Fig 1). (XLS)

S2 Table Variants in nucleotide and amino acids of TTV7 identified in individual patients with Kawasaki disease (KD) (spread sheet format for Fig 3). (XLS)
